# Aberrant Promoter Hypomethylation in CLL: Does It Matter for Disease Development?

**DOI:** 10.3389/fonc.2016.00182

**Published:** 2016-08-11

**Authors:** Garland Michael Upchurch, Staci L. Haney, Rene Opavsky

**Affiliations:** ^1^Fred and Pamela Buffett Cancer Center, Eppley Institute for Research in Cancer and Allied Diseases, University of Nebraska Medical Center, Omaha, NE, USA; ^2^Department of Genetics, Cell Biology, and Anatomy, University of Nebraska Medical Center, Omaha, NE, USA; ^3^Center for Leukemia and Lymphoma Research, University of Nebraska Medical Center, Omaha, NE, USA

**Keywords:** mouse models of cancer, chronic lymphocytic leukemia, DNA methyltransferases, hypomethylation, hematologic neoplasms, DNA methylation, leukemia, promoter methylation

## Abstract

Over the last 30 years, studies of aberrant DNA methylation in hematologic malignancies have been dominated by the primary focus of understanding promoter hypermethylation. These efforts not only resulted in a better understanding of the basis of epigenetic silencing of tumor suppressor genes but also resulted in approval of hypomethylating agents for the treatment of several malignancies, such as myelodysplastic syndrome and acute myeloid leukemia. Recent advances in global methylation profiling coupled with the use of mouse models suggest that aberrant promoter hypomethylation is also a frequent event in hematologic malignancies, particularly in chronic lymphocytic leukemia (CLL). Promoter hypomethylation affects gene expression and, therefore, may play an important role in disease pathogenesis. Here, we review recent findings and discuss the potential involvement of aberrant promoter hypomethylation in CLL.

## Introduction

Cytosine methylation of DNA is an epigenetic modification affecting gene transcription and the integrity of the mammalian genome. The three catalytically active DNA methyltransferases in mammalian cells are DNMT1, DNMT3A, and DNMT3B. These enzymes are responsible for establishment and maintenance of DNA methylation during normal development and during mitotic cell division. Promoter methylation typically results in transcriptional repression of genes and plays a role in various normal physiologic processes, such as differentiation and hematopoiesis (1, 2).

One of the main observations that contributed to an interest in studying this phenomenon came from studies that discovered that in virtually all types of cancer aberrantly increased methylation in gene promoters was associated with transcriptional inhibition (3). As a result, aberrant promoter hypermethylation resulting in silencing of tumor suppressor genes in cancer has been a major topic of numerous studies over the past 30 years. Such efforts not only resulted in identification of a number of epigenetically repressed tumor suppressor genes in hematologic malignancies, such as VHL, p16, and MLH1 (3), but also provided a conceptual approach to the treatment of cancer. Therefore, use of hypomethylating agents can be a valuable approach in anticancer therapy as reversal of DNA methylation may lead to reactivation of tumor suppressor genes and antagonize aberrant tumor proliferation and survival. Epigenetic studies in hematologic malignancies have consequently resulted in the approval of hypomethylating agents for the treatment of several malignancies, including myelodysplastic syndrome (MDS) and acute myeloid leukemia (AML). Numerous efforts are undergoing to refine hypomethylating agents and to test their efficacy in combination with other drugs targeting epigenetic changes.

The recent emergence of new high-resolution methylation profiling techniques, such as whole-genome bisulfite sequencing (WGBS), have revealed that the methylome of cancer cells frequently contains promoters that are hypomethylated relative to their normal cellular counterparts. Such aberrant hypomethylation is frequently accompanied by increased gene expression at differentially methylated loci. Whether or not such deregulated expression contributes to the initiation and progression of hematologic malignancies is currently unresolved and is actively under investigation.

Here, we will first review data obtained in mouse studies focusing on DNA methyltransferase loss of function in various hematologic malignancies. We will then discuss recent findings that strongly support the idea that aberrant promoter hypomethylation accompanied by gene re-expression may contribute to the development of chronic lymphocytic leukemia (CLL) in a causative manner.

## Role of DNA Methyltransferases in Mouse Normal and Malignant Hematopoiesis

Two activities for DNA methyltransferases are important in regard to genome-wide methylation patterns: *de novo* and maintenance. Early studies suggested that Dnmt3a and Dnmt3b are *de novo* enzymes capable of recognizing unmethylated DNA and catalyze the addition of a methyl group to cytosine (4). By contrast, Dnmt1 plays a role in maintenance of methylation profiles by recognizing methylated DNA and adding a methyl mark to the newly produced DNA strand during replication (5). The role of Dnmts in generating and maintaining methylation profiles in cancer is, however, less clear. In regard to gene promoters, from the most simplistic conceptual point of view, *de novo* methylation activity could be considered oncogenic because aberrant activity can generate new methylation marks in promoter sequences and inactivate genes with tumor suppressor functions. By contrast, maintenance methylation activity could be considered to be tumor suppressive because it can safeguard the integrity of the methylome and prevent inappropriate gene activation. However, a number of studies in which Dnmts were genetically inactivated have painted a rather different picture.

The most studied Dnmt in mouse hematopoiesis is Dnmt3a. Interest in studying the role of Dnmt3a in normal hematopoiesis was fueled by findings that in a number of hematologic malignancies of myeloid and T-cell origin Dnmt3a was mutated primarily in the catalytic domain, suggesting that methyltransferase activity is critical to prevent tumor development (6). Subsequently, several research groups have used mice to conditionally inactivate Dnmt3a in hematopoietic stem cells and early progenitors (HSPCs) by the *Mx1-Cre* transgene. Original reports suggested that inactivation of Dnmt3a results in a differentiation block and accumulation of progenitor cells; however, this phenotype was mainly observed upon serial bone marrow transplantation (7). The first report demonstrating that Dnmt3a plays a tumor suppressive role in the prevention of hematologic malignancy was published by Peters et al. (8), which showed that conditional inactivation of Dnmt3a in HSPCs using *E*μ*SR*α*-tTA;Teto-Cre;Dnmt3a^fl/fl^;Rosa26 LOXP^EGFP/EGFP^* quadruple transgenic mice (designated as *Dnmt3a*^Δ/Δ^ mice) results unexpectedly in the development of a CLL-like disease after 1 year’s time. This finding was surprising in view of a lack of genetic alterations in the DNMT3A locus in B-cell malignancies. Since this time, other groups have reported that Dnmt3a loss in HSPCs results in myriad types of malignancies, such as myeloproliferative disorders, AML, T-cell acute lymphoblastic leukemia (T-ALL), and B-cell acute lymphoblastic leukemia (B-ALL) (9, 10). Differences in phenotypes observed upon loss of Dnmt3a could stem from the different genetic background of mice used in these studies (Table 1) or the different properties of transgenes used to conditionally delete Dnmt3a alleles.

**Table 1:** **Summary of studies performed in mouse models in which Dnmts were ablated in hematopoietic cells**.

Transgenic model	Background	Phenotype	ONC/TS	Promoter hypomethylation	Reference
*Mx1-Cre; Dnmt1^fl/fl^*	Unknown	Bone marrow failure	N/A	Unknown	Bröske et al. (11)
*Dnmt1^chip^*^/−^	Unknown	T-cell lymphoma	TS	Unknown	Gaudet et al. (12)
*Dnmt1^chip^*^/−^; *retroviral Myc-Bcl2*	Unknown	Myeloid and T-lymphoid leukemia. Defects in B-cell development	N/A	Unknown	Bröske et al. (11)
*MLL-AF9; Mx1-Cre; Dnmt1^fl^*^/+^	C57Bl/6	AML	ONC	Unknown	Trowbridge et al. (13)
*E*μ*SR*α*-tTA; Teto-Cre; Teto-MYC; Dnmt1^fl/fl^; Rosa26 LOXP^EGFP/EGFP^*	FVB/N	T-cell lymphoma	ONC	Yes	Peters et al. (14)
*Mx1-Cre; Dnmt3a^fl/fl^*	C57Bl/6	HSC expansion and impaired differentiation	N/A	Yes	Challen et al. (7)
*Mx1-Cre; Dnmt3a^fl/fl^; Dnmt3b^fl/fl^*	C57Bl/6	HSC expansion and impaired differentiation	N/A	Yes	Challen et al. (15)
*E*μ*SR*α*-tTA; Teto-Cre; Dnmt3a^fl/fl^; Rosa26 LOXP^EGFP/EGFP^*	FVB/N	CLL, PTCL	TS	Yes	Peters et al. (8)
*E*μ*SR*α*-tTA; Teto-Cre; Teto-MYC; Dnmt3a^fl/fl^; Rosa26 LOXP^EGFP/EGFP^*	FVB/N	T-cell lymphoma	ONC	Yes	Haney et al. (16)
*Mx1-Cre; Dnmt3a^fl/fl^*	C57Bl/6	MDS, AML	TS	Unknown	Celik et al. (17)
*Mx1-Cre; Dnmt3a^fl/fl^*	C57Bl/6	MDS, AML, PMF, CMML, T-ALL, B-ALL, ETP	TS	Yes	Mayle et al. (9)
*Mx1-Cre; Dnmt3a^fl/fl^*	C57Bl/6	MDS/MPN	TS	Yes	Guryanova et al. (18)
*Mx1-Cre; Dnmt3a^fl/fl^; Kras^LSL G12D^*^/+^	C57Bl/6	AML	TS	Unknown	Chang et al. (19)
*Flt3*^+/^*^ITD^; Mx1-Cre; Dnmt3a^fl/fl^*	C57Bl/6	AML	TS	Yes	Meyer et al. (20)
*E*μ*SR*α*-tTA; Teto-Cre; Teto-MYC; Dnmt3b^fl/fl^; Rosa26 LOXP^EGFP/EGFP^*	FVB/N	T-cell lymphoma	TS	Yes	Hlady et al. (21)
*E*μ*SR*α*-tTA; Teto-Cre; Dnmt3b^fl/fl^; Rosa26 LOXP^EGFP/EGFP^*	FVB/N	No phenotype	N/A	Unknown	Hlady et al. (21)
*E*μ*-Myc;Dnmt3b*^+/−^	Unknown	B-cell lymphoma	TS	Unknown	Vasanthakumar et al. (22)
*Mx1-Cre;Dnmt3b^fl/fl^*	C57Bl/6	No phenotype	N/A	Unknown	Challen et al. (15)

*Unknown indicates that data were not currently available. ONC and TS stand for oncogene and tumor suppressor, respectively*.

*CLL, chronic lymphocytic leukemia; PTCL, peripheral T-cell lymphoma; HSC, hematopoietic stem cell; MDS, myelodysplastic syndrome; AML, acute myeloid leukemia; T-ALL, T-cell acute lymphoblastic leukemia; B-ALL, B-cell acute lymphoblastic leukemia; CMML, chronic myelomonocytic leukemia; PMF, primary myelofibrosis; ETP, early thymic progenitor acute lymphoblastic leukemia*.

In addition, Dnmt3a loss can collaborate with gain of function mutant c-kit^D814V^ to induce B-ALL, T-ALL, and mastocytosis with myeloid blasts (17), and with *Kras^G12D^*^/+^ to promote progression of juvenile and chronic myelomonocytic leukemia (CMML) (19). However, under some circumstances Dnmt3a has acted as an oncogene by promoting the development of hematologic malignancies. For example, upregulation of Dnmt3a promoted AML/ETO-induced leukemia through *de novo* hypermethylation (23) and a methylation-independent repressor function of Dnmt3a enhanced T-cell lymphomagenesis (24). Despite these last two examples, the overwhelming body of work in the field suggests that Dnmt3a acts as a tumor suppressor in various types of hematologic malignancies.

Due to the lack of genetic alterations in Dnmt3b in human malignancies, the role of this enzyme has been less well studied in mouse hematopoiesis. Loss of Dnmt3b does not appear to affect normal hematopoiesis but still has both overlapping and specific functions in HSPCs (21, 25). However, Dnmt3b has been shown to behave as a tumor suppressor gene in mouse models of Myc-induced T- and B-cell lymphomas (21, 22). Loss of Dnmt3b also accelerated development of CLL in *Dnmt3a*^Δ/Δ^ mice and also promoted the development of T-cell lymphomas (8).

Taken together, these data clearly show that Dnmt3a and Dnmt3b possess tumor suppressor functions in the prevention of the vast majority of hematologic malignancies (Table 1). These data further demonstrate that tumor development is accompanied by loss of methylation in promoter regions, suggesting that Dnmt3a and Dnmt3b may have a role in cancer-specific maintenance methylation of specific loci (Table 1). Given the large magnitude of promoter hypomethylation observed in various mouse hematologic malignancies (8, 9, 18, 26), it is possible that loss of Dnmt3a and Dnmt3b maintenance activity drives tumorigenesis due to the inappropriate expression of genes normally silenced.

In contrast to most studies on Dnmt3a and Dnmt3b, decreased levels of the *bona fide* maintenance enzyme Dnmt1 suppressed MYC-induced T-cell lymphomagenesis (14) (Table 1). In addition, Dnmt1 is critical for tumor maintenance in AML induced by MLL-AF9 overexpression or in B-cell leukemia induced by combined overexpression of Myc and Bcl2 (13, 27). However, there is also evidence that Dnmt1 can play a tumor suppressor role in prevention of T-cell lymphomas. *Dnmt1^chip^*^/−^ mice that carry a hypomorphic allele (*Dnmt1^chip^*) almost exclusively (91%) develop T-cell lymphomas (12). Thus, Dnmt1 seems to possess an oncogenic function by promoting cellular survival of both normal and tumor cells (Table 1).

Although evidence that aberrant promoter hypomethylation contributes to the development of hematologic malignancies is very limited at this point, we will discuss CLL as a disease in which deregulated methylation may matter the most for its initiation and progression.

## Promoter Hypomethylation in Human CLL

Chronic lymphocytic leukemia/small lymphocytic lymphoma (CLL/SLL) is an indolent low-grade lymphoproliferation of mature B-cells (28) and is the most common hematological malignancy in adults. The clinical course of CLL is highly variable, yet the majority of patients will progresses to a life-threatening condition requiring therapeutic intervention. Except for allogenic hematopoietic bone marrow transplantation, current treatments approved for CLL are not curative. An average of 45 somatic mutations is present in human CLL, with most genes mutated in <5% of cases (29). About one-third of cases did not have recurrent mutations, suggesting a high degree of genetic heterogeneity and no clear mutational drivers of CLL. Regarding the epigenetics of CLL, it is interesting to note that Dnmt3a and Dnmt3b were identified in the top 1% of underexpressed genes in human CLL (8, 30). Consistent with a role in disease initiation, global DNA hypomethylation and shortened telomeres were found to be significantly associated in early stage CLL tumors (Binet A) from untreated patients (31). Recent genome-wide methylation profiling revealed that the large-scale hypomethylation observed in CLL was not only limited to gene-bodies but also affected gene promoters (32). In fact, promoter hypomethylation was more than 10 times more frequent an event than promoter hypermethylation in both IGHV-unmutated (U-CLL, a more aggressive form of CLL) and IGHV-mutated CLL (M-CLL, a less aggressive form of CLL). U-CLL tumors contained greater than four times more hypomethylated promoters than M-CLL tumors, consistent with a possible role for promoter hypomethylation in disease progression. Additional studies using lower resolution methylation profiling also identified promoter hypomethylation in CLL, although it was less pronounced than promoter hypermethylation (33).

Altogether, these data provide evidence for extensive promoter hypomethylation in human CLL that may be involved in the pathogenesis of the disease.

## Aberrant Promoter Hypomethylation in Mouse CLL

A body of work using two mouse models has recently provided strong evidence that aberrant hypomethylation promotes CLL development. Transgenic *E*μ*-TCL1* is one of the first mouse models of CLL that was generated by using overexpression of the *TCL1* gene in mouse B cells (34). TCL1 protein acts as a coactivator of AKT serine–threonine kinase by transporting it to the nucleus that promotes cellular survival (35). An interest in understanding of TCL1 comes from findings that the gene is overexpressed in a number of human T-cell malignancies, including mature leukemias, T-cell prolymphocytic leukemia, and B-cell malignancies, such as Burkitt’s lymphoma (BL) and CLL (36, 37), suggesting its possible causative involvement in tumorigenesis. Indeed, *E*μ*-TCL1* mice developed CLL by 18 months of age that was characterized by splenomegaly and expansion of CD5^+^, IgM^+^ B1 cells in various organs, including the spleen, liver, and blood. The link to hypomethylation came from a recent study in which the authors reported that TCL1 binds to Dnmt3a and Dnmt3b and inhibits their activities (38). This raised the possibility that biochemical inhibition of Dnmts could be one activity though which TCL1 exerts its tumorigenic potential. Consistent with such idea, DNA methylation profiling revealed that pre-tumor B cells from *E*μ*-TCL1* mice showed a large degree of DNA hypomethylation. Although it had not been functionally proven at the time of this initial finding, independent studies have since demonstrated that either bi-allelic or mono-allelic loss of Dnmt3a in *Dnmt3a*^Δ/Δ^ and Dnmt3a^+/−^ mice is sufficient to induce CLL, indicating that Dnmt3a is a haploinsufficient tumor suppressor (8, 26). Importantly, both *Dnmt3a*^Δ/Δ^ and Dnmt3a^+/−^ CLL samples showed large-scale promoter hypomethylation strongly suggesting that aberrant promoter hypomethylation may play a role in disease development (26). Although loss of promoter hypermethylation does not necessarily result in gene re-expression and, in fact, such correlation in this study was only 10%, this still represented a large number (129) of inappropriately upregulated genes. By contrast, while promoter hypermethylation was detected in 118 genes it correlated with gene silencing of only one gene. Thus, deregulated changes in gene expression are more attributable to aberrant hypomethylation than hypermethylation. Such results potentially could be explained by another important observation that in mouse B-1a cells – the normal cellular counterpart of CLL in mice – two-third of promoters were >50% methylated (26). Such a baseline level of promoter methylation provides for the opportunity to lose rather than gain promoter methylation during tumor development, especially when methylation maintenance activity is compromised.

## Aberrantly Hypomethylated Genes with Potential Relevance to Human CLL

One of the clearest examples demonstrating that aberrant promoter hypomethylation plays a role in oncogenic transformation is the case of the TCL1 gene itself. Whereas in T-cell malignancies, an increased expression of TCL1 was linked to reciprocal translocations at chromosome segment 14q32.1, such genetic lesions were not observed in B-cell malignancies. Instead, promoter hypomethylation appears to induce overexpression of TCL1 in human CLL (36). Given that TCL1 overexpression results in the development of CLL in *E*μ*-TCL1* mice (34) this represents a clear demonstration of causative involvement of promoter hypomethylation in CLL development. Another example is the anomalous expression of the lipoprotein lipase (LPL) gene found to be expressed in human U-CLL, but not in M-CLL. Expression in U-CLL is dependent on a differentially hypomethylated LPL gene promoter, compared with M-CLL, and the presence of tumor micro-environmental signals to promote transcription (39). This finding implicates promoter hypomethylation as a facilitator of CLL and micro-environmental cross-talk.

A limited analysis of gene expression data revealed several genes that are upregulated in both *E*μ*-TCL1* and *Dnmt3a*^Δ/Δ^ CLL. This is consistent with the idea that Dnmt3a is biochemically inhibited in *E*μ*-TCL1* mice, which affects promoter methylation and this contributes to the development of CLL. Genes commonly upregulated between the two models include *Zbtb32, Slc7a7, Pstpip2, Pon3, Il5ra*, and *1810046K07Rik* (C11orf53). Interestingly, these genes are among the top 25 most overexpressed genes in the *E*μ*-TCL1* mouse model of CLL (40). Methylation status of their promoters in the *E*μ*-TCL1* model has not yet been carefully evaluated, but these genes have been shown to be aberrantly hypomethylated in *Dnmt3a*^Δ/Δ^ CLL. The functions of these genes in CLL development have not been evaluated to date. Therefore, we discuss here data for one protein coding gene – *Zbtb32* – and one gene encoding a non-coding RNA – *Pvt1*– that support their potential role in CLL.

Zbtb32 is a Zinc Finger and BTB Domain Containing 32 gene that was originally identified as a transcriptional repressor protein that interacts with several proteins including the Fanconi anemia group C protein and PLZF (41). Recently, *Zbtb32* was identified as a gene whose increased expression predicts whether healthy individuals without disease will develop CLL later in life (42). This raises the possibility that overexpression of ZBTB32 may causatively contribute to CLL development.

The *Pvt1* gene locus is a target of frequent tumorigenic translocations and retroviral insertions that result in overexpression of a long non-coding RNA that encodes several microRNAs that are overexpressed in hematological malignancies of B- and T-cell origin. For example, miR-1206 is overexpressed in human BLs, whereas miR-1204 is overexpressed in retrovirally induced T-cell lymphomas (43–45). Thus, aberrant hypomethylation of a single promoter may upregulate several micro RNAs possibly contributing to tumorigenesis.

## Future Directions

Given the large number of genes identified to be hypomethylated and overexpressed in mouse and human CLL, there is a clear potential that some of these genes are oncogenic drivers of CLL development. While convincing evidence for involvement of such genes in the pathogenesis of the disease is quite limited, a large body of data accumulated in recent years clearly points to such a possibility. A conceptual justification for such efforts is not limited to just an understanding of basic mechanisms governing cellular transformation but might also have direct implication for targeted therapies. Frequently, oncogenes that initiate tumor development are also important for maintenance of tumor phenotype. Therefore, their targeting may prove to be useful in anti-neoplastic therapy. Consequently, major efforts will likely be undertaken in a future to identify proto-oncogenes silenced in normal cells by promoter methylation that become de-methylated during tumor initiation/progression and functionally contributing to cellular transformation. Such efforts will likely not be limited to CLL only but will include other types of hematologic malignancies and potentially solid tumors.

## Author Contributions

GU and RO discussed, designed, and conceptualized the review article. GU, RO, and SH wrote, edited, and revised the review article. Primary literature review was performed by GU and RO. SH and GU constructed Table 1.

## Conflict of Interest Statement

The authors declare that the research was conducted in the absence of any commercial or financial relationships that could be construed as a potential conflict of interest. The reviewer BW and handling editor declared their shared affiliation, and the handling editor states that the process nevertheless met the standards of a fair and objective review.

## References

[1] McCabe MT Brandes JC Vertino PM. Cancer DNA methylation: molecular mechanisms and clinical implications. Clin Cancer Res (2009) 15:3927–37. 10.1158/1078-0432.CCR-08-2784 19509173 PMC2715155

[2] Celik H Kramer A Challen GA. DNA methylation in normal and malignant hematopoiesis. Int J Hematol (2016) 103(6):617–26. 10.1007/s12185-016-1957-7 26943352

[3] Herman JG Baylin SB Gene silencing in cancer in association with promoter hypermethylation. N Engl J Med (2003) 349:2042–54. 10.1056/NEJMra023075 14627790

[4] Okano M Bell DW Haber DA Li E. DNA methyltransferases Dnmt3a and Dnmt3b are essential for de novo methylation and mammalian development. Cell (1999) 99:247–57. 10.1016/S0092-8674(00)81656-6 10555141

[5] Robert MF Morin S Beaulieu N Gauthier F Barsalou A MacLeod AR DNMT1 is required to maintain CpG methylation and aberrant gene silencing in human cancer cells. Nat Genet (2002) 33:61–5. 10.1038/ng1068 12496760

[6] Yang L Rau R Goodell MA. DNMT3A in haematological malignancies. Nat Rev Cancer (2015) 15:152–65. 10.1038/nrc3895 25693834 PMC5814392

[7] Challen GA Sun D Jeong M Luo M Jelinek J Berg JS Dnmt3a is essential for hematopoietic stem cell differentiation. Nat Genet (2011) 44:23–31. 10.1038/ng.1009 22138693 PMC3637952

[8] Peters SL Hlady RA Opavska J Klinkebiel D Pirruccello SJ Talmon GA Tumor suppressor functions of Dnmt3a and Dnmt3b in the prevention of malignant mouse lymphopoiesis. Leukemia (2014) 28:1138–42. 10.1038/leu.2013.364 24292811

[9] Mayle A Yang L Rodriguez B Zhou T Chang E Curry CV Dnmt3a loss predisposes murine hematopoietic stem cells to malignant transformation. Blood (2015) 125:629–38. 10.1182/blood-2014-08-594648 25416277 PMC4304108

[10] Guryanova OA Lieu YK Garrett-Bakelman FE Spitzer B Glass JL Shank K Dnmt3a regulates myeloproliferation and liver-specific expansion of hematopoietic stem and progenitor cells. Leukemia (2016) 5:133–42. 10.1038/leu.2015.358 26710888 PMC4856586

[11] Bröske AM Vockentanz L Kharazi S Huska MR Mancini E Scheller M DNA methylation protects hematopoietic stem cell multipotency from myeloerythroid restriction. Nat Genet (2009) 41:1207–15. 10.1038/ng.463 19801979

[12] Gaudet F Hodgson JG Eden A Jackson-Grusby L Dausman J Gray JW Induction of tumors in mice by genomic hypomethylation. Science (2003) 300:489–92. 10.1126/science.1083558 12702876

[13] Trowbridge JJ Sinha AU Zhu N Li M Armstrong SA Orkin SH. Haploinsufficiency of Dnmt1 impairs leukemia stem cell function through derepression of bivalent chromatin domains. Genes Dev (2012) 26:344–9. 10.1101/gad.184341.111 22345515 PMC3289882

[14] Peters SL Hlady RA Opavska J Klinkebiel D Novakova S Smith LM Essential role for Dnmt1 in the prevention and maintenance of MYC-induced T-cell lymphomas. Mol Cell Biol (2013) 33:4321–33. 10.1128/MCB.00776-13 24001767 PMC3811897

[15] Challen GA Sun D Mayle A Jeong M Luo M Rodriguez B Dnmt3a and Dnmt3b have overlapping and distinct functions in hematopoietic stem cells. Cell Stem Cell (2014) 15(3):350–64. 10.1016/j.stem.2014.06.018 25130491 PMC4163922

[16] Haney SL Hlady RA Opavska J Klinkebiel D Pirruccello SJ Dutta S Methylation-independent repression of Dnmt3b contributes to oncogenic activity of Dnmt3a in mouse MYC-induced T-cell lymphomagenesis. Oncogene (2015) 34(43):5436–46. 10.1038/onc.2014.472 25639876 PMC4533871

[17] Celik H Mallaney C Kothari A Ostrander EL Eultgen E Martens A Enforced differentiation of Dnmt3a-null bone marrow leads to failure with c-Kit mutations driving leukemic transformation. Blood (2015) 125:619–28. 10.1182/blood-2014-08-594564 25416276 PMC4304107

[18] Guryanova OA Lieu YK Garrett-Bakelman FE Spitzer B Glass JL Shank K Dnmt3a regulates myeloproliferation and liver-specific expansion of hematopoietic stem and progenitor cells. Leukemia (2016) 30(5):1133–42. 10.1038/leu.2015.358 26710888 PMC4856586

[19] Chang YI You X Kong G Ranheim EA Wang J Du J Loss of Dnmt3a and endogenous Kras(G12D/+) cooperate to regulate hematopoietic stem and progenitor cell functions in leukemogenesis. Leukemia (2015) 9:1847–56. 10.1038/leu.2015.85 25801914 PMC4558337

[20] Meyer SE Qin T Muench DE Masuda K Venkatasubramanian M Orr E DNMT3A Haploinsufficiency Transforms FLT3ITD Myeloproliferative Disease into a Rapid, Spontaneous, and Fully Penetrant Acute Myeloid Leukemia. Cancer Discov (2016) 6(5):501–15. 10.1158/2159-8290.CD-16-0008 27016502 PMC4861898

[21] Hlady RA Novakova S Opavska J Klinkebiel D Peters SL Bies J Loss of Dnmt3b function upregulates the tumor modifier Ment and accelerates mouse lymphomagenesis. J Clin Invest (2012) 122(1):163–77. 10.1172/JCI57292 22133874 PMC3248285

[22] Vasanthakumar A Lepore JB Zegarek MH Kocherginsky M Sing M Davis EM Dnmt3b is a haploinsufficient tumor suppressor gene in Myc-induced lymphomagenesis. Blood (2013) 121:2059–63. 10.1182/blood-2012-04-421065 23315164 PMC3596965

[23] Gao XN Yan F Lin J Gao L Lu XL Wei SC AML1/ETO cooperates with HIF1α to promote leukemogenesis through DNMT3a transactivation. Leukemia (2015) 29:1730–40. 10.1038/leu.2015.56 25727291

[24] Haney SL Hlady RA Opavska J Klinkebiel D Pirruccello SJ Dutta S Methylation-independent repression of Dnmt3b contributes to oncogenic activity of Dnmt3a in mouse MYC-induced T-cell lymphomagenesis. Oncogene (2015) 34:5436–46. 10.1038/onc.2014.472 25639876 PMC4533871

[25] Challen GA Sun D Mayle A Jeong M Luo M Rodriguez B Dnmt3a and Dnmt3b have overlapping and distinct functions in hematopoietic stem cells. Cell Stem Cell (2014) 15:350–64. 10.1016/j.stem.2014.06.018 25130491 PMC4163922

[26] Haney SL Upchurch GM Opavska J Klinkebiel D Hlady RA Suresh A Promoter hypomethylation and expression is conserved in mouse chronic lymphocytic leukemia induced by decreased or inactivated Dnmt3a. Cell Rep (2016) 15:1190–201. 10.1016/j.celrep.2016.04.004 27134162 PMC4864108

[27] Bröske AM Vockentanz L Kharazi S Huska MR Mancini E Scheller M DNA methylation protects hematopoietic stem cell multipotency from myeloerythroid restriction. Nat Genet (2009) 41(11):1207–15. 10.1038/ng.463 19801979

[28] Fabbri G Dalla-Favera R. The molecular pathogenesis of chronic lymphocytic leukaemia. Nat Rev Cancer (2016) 16:145–62. 10.1038/nrc.2016.8 26911189

[29] Quesada V Conde L Villamor N Ordóñez GR Jares P Bassaganyas L Exome sequencing identifies recurrent mutations of the splicing factor SF3B1 gene in chronic lymphocytic leukemia. Nat Genet (2011) 44:47–52. 10.1038/ng.1032 22158541

[30] Haferlach T Kohlmann A Wieczorek L Basso G Kronnie GT Béné MC Clinical utility of microarray-based gene expression profiling in the diagnosis and subclassification of leukemia: report from the International Microarray Innovations in Leukemia Study Group. J Clin Oncol (2010) 28:2529–37. 10.1200/JCO.2009.23.4732 20406941 PMC5569671

[31] Hoxha M Fabris S Agnelli L Bollati V Cutrona G Matis S Relevance of telomere/telomerase system impairment in early stage chronic lymphocytic leukemia. Genes Chromosomes Cancer (2014) 53(7):612–21. 10.1002/gcc.22171 24706380

[32] Kulis M Heath S Bibikova M Queirós AC Navarro A Clot G Epigenomic analysis detects widespread gene-body DNA hypomethylation in chronic lymphocytic leukemia. Nat Genet (2012) 44:1236–42. 10.1038/ng.2443 23064414

[33] Pei L Choi JH Liu J Lee EJ McCarthy MB Wilson JM Genome-wide DNA methylation analysis reveals novel epigenetic changes in chronic lymphocytic leukemia. Epigenetics (2012) 7(6):567–78. 10.4161/epi.20237 22534504 PMC3398986

[34] Bichi R Shinton SA Martin ES Koval A Calin GA Cesari R Human chronic lymphocytic leukemia modeled in mouse by targeted TCL1 expression. Proc Natl Acad Sci U S A (2002) 99:6955–60. 10.1073/pnas.102181599 12011454 PMC124510

[35] Laine J Künstle G Obata T Sha M Noguchi M. The protooncogene TCL1 is an Akt kinase coactivator. Mol Cell (2000) 6(2000):395–407. 10.1016/S1097-2765(00)00039-3 10983986

[36] Yuille MR Condie A Stone EM Wilsher J Bradshaw PS Brooks L TCL1 is activated by chromosomal rearrangement or by hypomethylation. Genes Chromosomes Cancer (2001) 30:336–41. 10.1002/gcc.1099 11241786

[37] Herling M Patel KA Khalili J Schlette E Kobayashi R Medeiros LJ TCL1 shows a regulated expression pattern in chronic lymphocytic leukemia that correlates with molecular subtypes and proliferative state. Leukemia (2006) 20:280–5. 10.1038/sj.leu.2404017 16341048

[38] Palamarchuk A Yan PS Zanesi N Wang L Rodrigues B Murphy M Tcl1 protein functions as an inhibitor of de novo DNA methylation in B-cell chronic lymphocytic leukemia (CLL). Proc Natl Acad Sci U S A (2012) 109:2555–60. 10.1073/pnas.1200003109 22308499 PMC3289317

[39] Abreu C Moreno P Palacios F Borge M Morande P Landoni AI Methylation status regulates lipoprotein lipase expression in chronic lymphocytic leukemia. Leuk Lymphoma (2013) 54(8):1844–8. 10.3109/10428194.2013.796057 23614796

[40] Nganga VK Palmer VL Naushad H Kassmeier MD Anderson DK Perry GA Accelerated progression of chronic lymphocytic leukemia in Eμ-TCL1 mice expressing catalytically inactive RAG1. Blood (2013) 121:3855–66. 10.1182/blood-2012-08-446732 23502221 PMC3700393

[41] Hoatlin ME Zhi Y Ball H Silvey K Melnick A Stone S A novel BTB/POZ transcriptional repressor protein interacts with the Fanconi anemia group C protein and PLZF. Blood (1999) 94:3737–47. 10572087

[42] Chadeau-Hyam M Vermeulen RC Hebels DG Castagné R Campanella G Portengen L Prediagnostic transcriptomic markers of chronic lymphocytic leukemia reveal perturbations 10 years before diagnosis. Ann Oncol (2014) 25:1065–72. 10.1093/annonc/mdu056 24558024 PMC4366593

[43] Beck-Engeser GB Lum AM Huppi K Caplen NJ Wang BB Wabl M. Pvt1-encoded microRNAs in oncogenesis. Retrovirology (2008) 5:4. 10.1186/1742-4690-5-4 18194563 PMC2257975

[44] Guan Y Kuo WL Stilwell JL Takano H Lapuk AV Fridlyand J Amplification of PVT1 contributes to the pathophysiology of ovarian and breast cancer. Clin Cancer Res (2007) 13:5745–55. 10.1158/1078-0432.CCR-06-2882 17908964

[45] Huppi K Volfovsky N Runfola T Jones TL Mackiewicz M Martin SE The identification of microRNAs in a genomically unstable region of human chromosome 8q24. Mol Cancer Res (2008) 6:212–21. 10.1158/1541-7786.MCR-07-0105 18314482

